# Do online social media cut through the constraints that limit the size of offline social networks?

**DOI:** 10.1098/rsos.150292

**Published:** 2016-01-20

**Authors:** R. I. M. Dunbar

**Affiliations:** Department of Experimental Psychology, University of Oxford, South Parks Road, Oxford OX1 3UD, UK

**Keywords:** network size, social networking sites (SNSs), social brain hypothesis, egocentric networks

## Abstract

The social brain hypothesis has suggested that natural social network sizes may have a characteristic size in humans. This is determined in part by cognitive constraints and in part by the time costs of servicing relationships. Online social networking offers the potential to break through the glass ceiling imposed by at least the second of these, potentially enabling us to maintain much larger social networks. This is tested using two separate UK surveys, each randomly stratified by age, gender and regional population size. The data show that the size and range of online egocentric social networks, indexed as the number of Facebook friends, is similar to that of offline face-to-face networks. For one sample, respondents also specified the number of individuals in the inner layers of their network (formally identified as support clique and sympathy group), and these were also similar in size to those observed in offline networks. This suggests that, as originally proposed by the social brain hypothesis, there is a cognitive constraint on the size of social networks that even the communication advantages of online media are unable to overcome. In practical terms, it may reflect the fact that real (as opposed to casual) relationships require at least occasional face-to-face interaction to maintain them.

## 1. Introduction

Thanks to the Internet, the past decade has witnessed a dramatic revolution in our social world. By providing novel channels that allow us to communicate with individuals that we would otherwise have difficulty meeting face to face, the Internet has made it possible to service existing relationships as well as meet new individuals more efficiently and on a wider geographical scale. Given the extensive use of social media, the question as to whether Internet-based social networking sites (SNSs) have a positive or negative impact on social relationships has been much debated [1,2]. Cyberpessimists (e.g. [3–7]) have argued that the Internet has detrimental effects on our social life. In contrast, cyberoptimists (e.g. [8,9]) have insisted that the effects have been beneficial in many different ways (for reviews, see [10,11]).

There is some evidence to suggest that one of the motivations for using social media among teenagers is to extend their range of social contacts [12]. Indeed, it has specifically been claimed that those who are more socially competent use social media to expand their network of friendships, thereby increasing their social capital [13–16]. However, young children, and to some extent teenagers, are relatively poor at judging relationship quality: very young children, for example, commonly mistake a desire to form friendships on their part with the assumption that such friendships are reciprocated [17]. Adults tend to be more attuned to the nuances in different types of relationship and are less prone to signing up to ‘friending’ requests without considering the nature of the relationship involved. They may thus provide a better test of the hypothesis than the teenagers who have been the focus of most social media research.

One specific respect in which the Internet has been held to change our social world is the size of our social networks. On the basis of a projection from an equation relating social community size to neocortex volume in apes, it had previously been suggested that there is a natural group size for humans [18], and this has been validated against hunter–gatherer community sizes [19,20] as well as the sizes of offline personal social networks (egocentric networks) in two European populations [21,22]. This limit is thought to arise from a combination of a cognitive constraint (the product of the relationship with neocortex size known as the social brain hypothesis (SBH) [18,23]) and a time constraint associated with the costs of servicing relationships [24,25]. Implicit evidence for a potential cognitive constraint has been provided by a number of neuroimaging studies which show that individual variation in adult social network size correlates with the volume of core areas in the neocortex (notably those regions of the prefrontal and temporal lobes) that are associated with the ‘theory of mind’ network in humans [26–29], and this also seems to be true of monkeys [30].

An important feature of natural social networks in both humans [19,20,25] and non-human primates [31] is that they are structured into a distinctive series of hierarchically inclusive layers that have a natural scaling ratio of approximately 3. These layers reflect both interaction frequencies and, at least in humans, emotional closeness [22,25,31]. In humans, these layers have values that approximate 5, 15, 50 and 150, and extend beyond this in at least two further layers to 500 and 1500 [32]. The first three layers have been identified in several online datasets [33] and, at least in humans, appear to be a consequence of a constraint on available social time [34] combined with a relationship between time invested in a relationship and its quality (as rated in terms of emotional closeness) [25,35,36]. The two outermost layers (at 500 and 1500) correspond, respectively, to acquaintances (people we would not consider as personal friends or family, but know well enough to have a conversation with) and to the number of faces we can put names to.

It has been suggested that, even if this limit on personal network size exists in the face-to-face world, the rise of online SNSs has circumvented at least some of these constraints and has thus allowed us to increase dramatically the number of people we can have as friends [9,37–39]. Because there are significant limits on the number of people we can talk to at any one time in the offline world [40–42] as well as on the amount of time we have available for social interaction [25,43], there is inevitably a limit on the size of our egocentric social networks when relationships require time investment. In contrast, there are no limits to the number of people who can read our posts, and SNSs might thus allow us to cut through this constraint imposed by face-to-face interaction. Being able to interact with many individuals at the same time could in principle allow us to increase social network size dramatically.

Although many recent studies have undertaken comparisons between online and offline networks, almost all have focused on the small number of strong ties [44] that individuals have [45–50]. The definitions used in sampling networks in these studies have often been so restrictive that the typical network size they reveal has been in the order of five individuals (and never more than 20), suggesting that they are sampling just the two innermost core layers of personal egocentric networks (typically five and 12–15 individuals, respectively [25,34]). These sampling strategies thus ignore the fact that an individual’s social network (defined as all meaningful relationships) is considerably larger than this, and typically in the order of 100–200 individuals.

In addition, these studies have, with very few exceptions, sampled students (and, in many cases, teenagers), heavy SNS users or members of other specialized communities [2,51], and so cannot be considered representative of the wider population. The few studies that have undertaken large-scale randomized samples representative of the population at large [2,48] have focused only on the innermost layers of the network and have had quite modest sample sizes (*N*≈1000 Internet users) by the standards of sociological sampling. Two attempts to examine the size of extended online social networks with seriously large samples have considered communities formed among twitterati (i.e. followers of a particular twitter account) [52] and scientific email communities [53]. Both claimed evidence for a natural community size between 100 and 200, but neither of these can really be considered conventional everyday social communities in any meaningful sense.

This study tests the claim that online social environments allow us to significantly increase the size of our social networks using two large structured random samples of the UK population and the number of friends listed on Facebook as the test metric. These data constitute the first attempt to determine the natural limit on network size using unbiased, randomized, stratified sampling of a national population. As such, this study is the first real attempt to test whether online social media do allow us to increase the size of our social networks.

## 2. Material and methods

The data derive from two samples commissioned from the panel provider OnePoll by the WildCard agency on behalf of the Thomas J. Fudge’s company, sampled from OnePoll’s large in-house panel. The samples were carried out in the first week of April 2015 and the third week of May 2015, respectively. Each sample was a nationally structured random sample of adults aged 18–65 years distributed proportionally to age, sex and regional population across the UK.

Sample 1 included 2000 adults (mean age 39 years; 45.2% male), focused on people’s use of, and satisfaction with, online social media, with adults who ‘made regular use of social media’ as the sampling criterion. A total of 85.4% of respondents declared that they checked social media every day; 51% declared that they had never deleted their social media profile. Only 12.3% of the entire sample declared that, when they had deleted a profile, they had lasted more than two weeks before signing back in, with 4% declaring that they had lasted more than a year. Sample 2 included 1375 adults (mean age = 37.4 years; 39.1% male) and sampled professional adults who worked full time at 9.00 to 17.00 weekday jobs and had attended business meetings on behalf of their employer. In this sample, respondents were not necessarily social media users, and Sample 2 thus might be seen as being more representative of the general population. Each sample included approximately 30 questions related to use of online media (Sample 1) and social behaviour in relation to management meetings (Sample 2), with network size questions included as part of these.

In both cases, subjects were asked to state, on a 14-point (Sample 1) or 16-point (Sample 2) categorical scale ranging from 0 to 1000+, how many friends they had on Facebook (electronic supplementary material, tables S1 and S8). Categorical answers were used rather than asking for actual numbers of Facebook friends because, in large-scale sampling, it is important to maintain respondent interest and focus, and not to distract them by requiring them to break off and open new windows. Respondents in Sample 1 were also asked to state how many of these individuals they considered to be close friends (on a 12-point categorical scale ranging from 0 to 100+: electronic supplementary material, table S4) and how many individuals they would ‘consider going to for advice or sympathy in times of great emotional or other distress’ (on a 7-point categorical scale ranging between 0 and 16+: electronic supplementary material, table S5). These correspond to the two innermost circles of the egocentric social network, the sympathy group (normatively approx. 15 individuals) and the support clique (normatively approx. 5 individuals) [25,54].

## 3. Results

**Figure 1.**
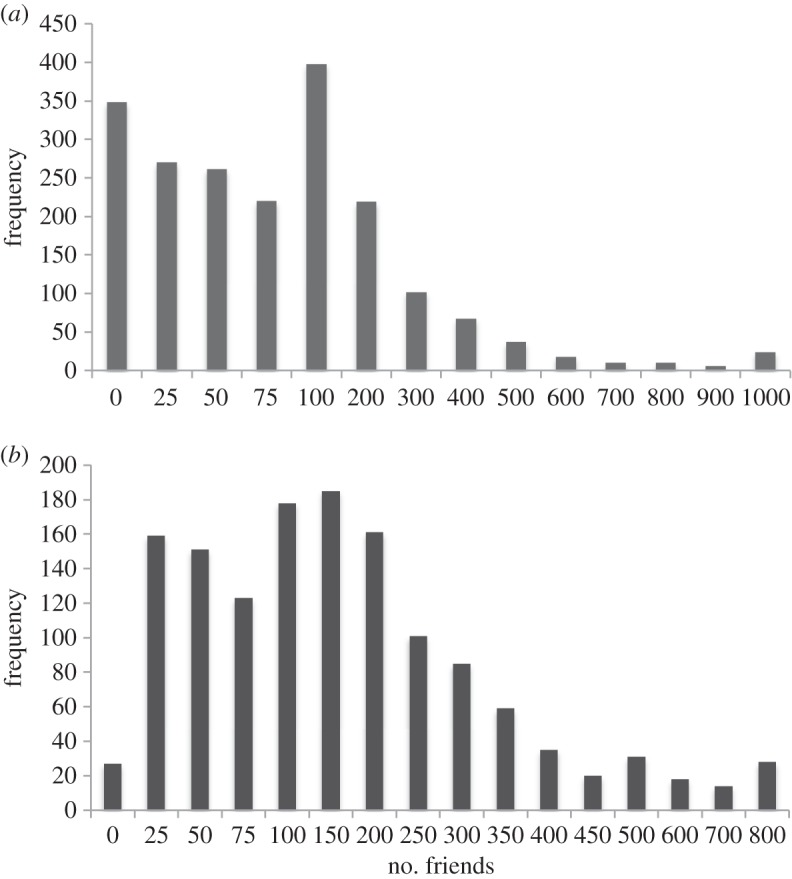
Distribution of network size for (*a*) Sample 1 (social network users: *N*=2000) and (*b*) Sample 2 (business employees: *N*=1375).

Figure 1 plots the distribution of total number of friends for the two samples. The mean number of friends is 155.2 and 182.8 in the two samples, respectively. Neither of these differs significantly from the value of 150 (95% CI = 100–200) predicted by the SBH [18] (*z*=0.20, *p*=0.842; *z*=1.29, *p*=0.198, respectively). (If we exclude those who responded 0 in Sample 2, the mean increases to 186.5, but the conclusion is unchanged.) As has been noted in all previous studies reporting network size, both distributions have long tails to the right, but have marked modes around 150. Although the two distributions differ significantly from each other (*χ*^2^=191.6, d.f.=9,*p*<0.0001), this is in fact mainly due to the high frequency of 0 scores in Sample 2. Given the difference in sampling criteria (regular social media users versus business professionals), this is perhaps hardly surprising. Discounting the 0 category, the two distributions in fact correlate closely across size categories (*r*=0.909,*N*=11, *p*<0.001; the slope does not differ significantly from *β*=1, *t*_9_=0.293, *p*=0.776).

In both cases, women list significantly more friends than men (Sample 1: 165.5 versus 145.0; Sample 2: 196.2 versus 156.6; *χ*^2^=33.3 and *χ*^2^=56.1, respectively; d.f.=8, *p*<0.0001). Network size also varies significantly by decadal age class: in each sample, mean network size is negatively related to age class, with younger age classes having larger online networks than older age classes (electronic supplementary material, tables S3 and S9; *r*=−0.972, *p*=0.006, and *r*=−0.973, *p*=0.005; *N*=5 in both cases). Both distributions differ significantly across age classes (*χ*^2^=436.7, d.f.=28, and *χ*^2^=352.1, d.f.=24; *p*<0.0001). Partitioning *χ*^2^ indicates that, in both samples, each age class differs significantly from the others (*χ*^2^≥54.2 and *χ*^2^≥21.5, respectively, *p*<0.005). Note that the mean values for each age class are almost identical in the two samples (electronic supplementary material, figure S1: Pearson’s *r*=0.993, standardized *β*=0.993,*N*=5,*p*=0.001).

**Figure 2.**
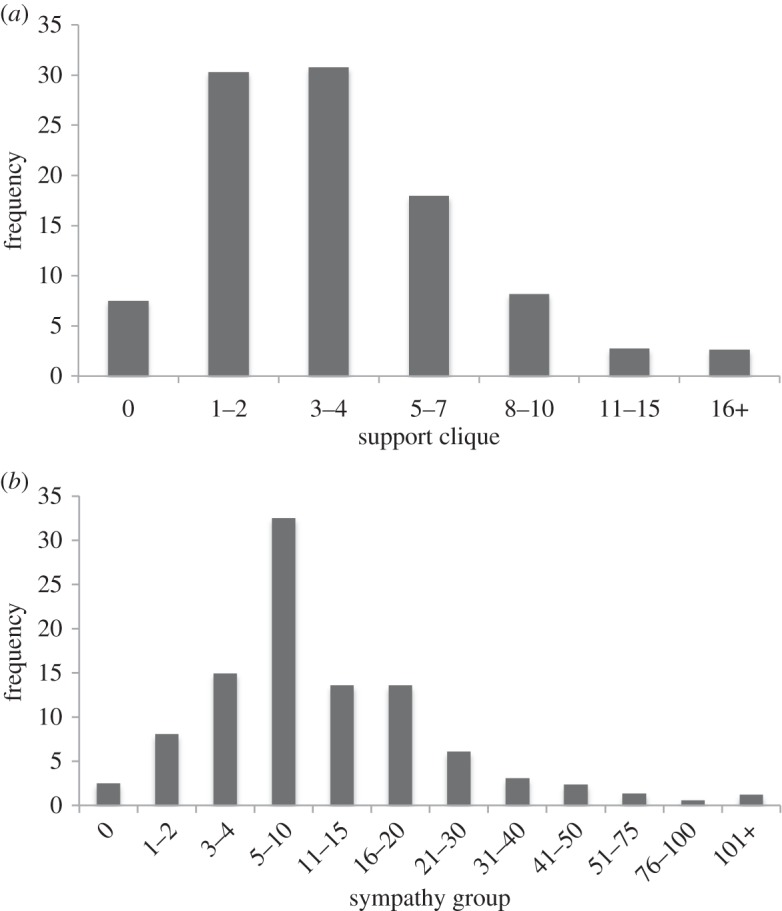
Distribution of (*a*) support clique size and (*b*) sympathy group size for Sample 1 (*N*=2000).

On average, respondents in Sample 1 considered that only 27.6% of their Facebook friends could be considered ‘genuine’ (i.e. close) friends, with a strong modal value between 0 and 10% (electronic supplementary material, table S2 and figure S2). These respondents were also asked more explicit questions about the number of close friends they had. Figure 2 plots the distribution of the support clique and the number of close friends (sympathy group) for Sample 1. The mean values are 4.1 and 13.6, respectively, for the support clique (friends on whom you would depend for emotional/social support in times of crisis) and sympathy group (close friends). Neither mean is significantly different from the values of 3.8±2.29 and 11.3±6.19 given for these two grouping layers by Hill & Dunbar [21] based on the literature (standardized deviates: *z*=0.13, *p*=0.897, and *z*=0.37, *p*=0.711, respectively) or from the generic values of 5 and 15 identified by Zhou *et al*. [19] (*z*=0.39,*p*=0.694, and *z*=0.23, *p*=0.818, respectively). It is noteworthy that the mean size of the support clique and sympathy group hardly vary at all with age (electronic supplementary material, tables S5 and S7) (*r*=−0.837, *p*=0.077, and *r*=−0.042, *p*=0.947, respectively; *N*=5). The distribution of support clique values does not differ across decadal age classes (*χ*^2^=9.79, d.f.=20, *p*=0.972). Although the distributions do differ significantly across age classes for the sympathy group data (*χ*^2^=45.76, d.f.=24, *p*=0.0047), most of the deviations of observed from expected are in fact modest and rather inconsistently distributed.

## 4. Discussion

These data allow us to draw two conclusions. First, they confirm, using two separate, large, nationally stratified random samples, that the typical egocentric network size for adult humans is similar to that predicted by the SBH. The selection criteria for the two samples were quite different and could have yielded very different outcomes. In fact, both estimates of network size were well within the 95% confidence limits around the predicted value from the SBH (roughly 100–200 [18]). As with more conventional offline networks, there is considerable variance around the mean values. In offline networks, this variance is due to personality differences (extroverts have larger networks than introverts [55]), gender (women have larger networks than men [56]) and, as we show here, age (electronic supplementary material, tables S3 and S9).

Sample 1 also confirms the existence and size of the two innermost layers of the social network, now conventionally identified as the support clique and the sympathy group [25]. Numerically, the innermost layer (five individuals) has been found in almost every online and offline study that has sampled network sizes, where estimates have varied from 4.0 to 9.0 in online samples [2,46,48,57] and 2.1 to 7.4 in offline studies [46,48,54,56,58–65]. The 15-layer has likewise been widely identified, with online values varying between 11.2 and 20.9 [2,45,48] and offline between 9.0 and 20.0 [28,48,54,56,66–68]. In the present sample, the mean values for these two layers (4.1 and 13.6) fit comfortably within these ranges.

The second main finding is that the two samples provide a direct test of whether online SNSs allow individuals to have larger social networks than is possible offline because SNSs allow one or more of the constraints that limit offline social network size to be circumvented. The results clearly suggest that they do not. This result concurs with previous findings for a much smaller sample (*N*=117) which suggested that heavy users of online social media do not have larger offline social networks than casual users, even though more of these may appear online for heavy users [69]. Previous studies that have looked at this question have typically focused exclusively on the innermost (5) network layer, and this study is the first to test the full social network.

We might argue that the long tails to the right provide evidence that, even if most do not, some people can maintain larger networks online (i.e. a small number of people really do have large numbers of listed friends). If so, it would seem that this does not apply universally: only 14.2% of respondents in Sample 1 listed more than 300 friends (i.e. significantly more than the average offline network) and only 13.4% of those in Sample 2 did so. Since respondents were not asked to specify the quality of their relationships with individual alters, we cannot say whether the extra members in these larger networks really are additional high-quality relationships or simply individuals that would normally be included in the circle of acquaintances that forms a further layer stretching out to 500 individuals in the offline world [19,25]. In most online platforms, these are all formally subsumed under the single category ‘friends’, whereas in the offline world we would naturally distinguish between friends and acquaintances of different emotional quality (and may even make that distinction informally for ourselves online).

In fact, analyses of traffic in online environments such as Facebook and Twitter reproduce rather faithfully both the nested structure of the inner layers of offline networks and their typical interaction frequencies [33]. Taken together with the fact that, in this study, the sizes of the two inner friendship circles did not differ from those previously identified in offline samples (figure 2), this suggests that it is most likely failure to differentiate relationships of different quality in the outermost layers that leads to the impression of large numbers of online ‘friends’ (see also [2]). Respondents who had unusually large networks did not increase the numbers of close friendships they had, but rather added more loosely defined acquaintances into their friendship circle simply because most social media sites do not allow one to differentiate between these layers (see also below).

The data in the two samples confirm previous findings of a small but consistent difference in network size between the sexes (with females generally having larger networks at any given layer than males) [21,28,54,56]. In both samples in this study, women had significantly larger networks than men, though the differences remain within the natural range of variation in egocentric social network size. This is in line with previous studies, which have suggested that women have more friends than men at least in the innermost layers (though, again, the absolute differences are numerically modest, and sometimes not significant) [28,56,70]. This may be related to women’s greater social skills, as reflected in their higher scores on the kinds of cognitive tasks (e.g. mentalizing) that are thought to underpin social relationships [56,71].

The data also highlight a strong age effect in complete network size: younger respondents (18–24 year olds) had significantly larger networks than older respondents (55+ year olds) (electronic supplementary material, tables S3 and S9). Rosen *et al.* [57] report a mean network size of 249 for a sample with a mean age of 19.5 years, which is close to the mean of 282 found in the 18–24-year-old age group in this study (electronic supplementary material, table S3). It is noteworthy that this is also close to the ‘optimal’ number of online friends that maximizes social attractiveness: in a study of students (mean age 20.2 years), Tong *et al.* [72] found that ratings for attractiveness of fictitious profiles peaked at profiles which listed 302 friends (in a range covering 102–902 in steps of 200). This may reflect well-known differences in how teenagers (in particular) and older adults use social media, with younger individuals using it in a more exploratory way to meet new people [12].

Note, however, that there is no equivalent age effect for the inner two layers of the network (electronic supplementary material, tables S5 and S7), suggesting that the age effect applies only in the outermost layer(s) of more casual friendships. Even so, this age effect contrasts with findings from offline networks, where younger respondents tend to have significantly smaller social networks than older adults [21,24,35]. A likely explanation for this difference probably lies in the fact that SNSs typically encourage promiscuous ‘friending’ of individuals who often have very tenuous links to ego (X is a friend [or friend-of-friend-of-a-friend] of Y, so would you like to befriend them?). Given that children are less discriminating than adults in defining friendships [17], this may cause younger people in general to respond more positively to these invitations. In addition, teenagers and young adults are in a period of their lives when searching for new friendship (and especially romantic) opportunities is a particularly important part of the natural life cycle; this may encourage individuals to establish many weak links with alters as a means of testing out the opportunities available to them.

It is perhaps worth noting that there has been a notable tendency for teenagers to move away from using Facebook as a social environment and to make use of media like Snapchat, WeChat, Vine, Flickr and Instagram instead [73], with Facebook being reserved mainly for managing social arrangements. It is not yet entirely clear what has driven this, but the fact that Facebook is too open to view by others seems to have been especially important [74,75]. Teenagers have much smaller offline social networks than adults [24,76], and forcing them to enlarge their network with large numbers of anonymous ‘friends-of-friends’ may place significant strain on their ability to manage their networks. Thus, this trend towards more private social media may actually confirm the claim being made here—that open-ended social media do not in fact allow us to increase the sizes of our social circles beyond that imposed by the SBH and the constraints of everyday offline interaction.

The fact that social networks remain about the same size despite the communication opportunities provided by social media suggests that the constraints that limit face-to-face networks are not fully circumvented by online environments. Instead, it seems that online social networks remain subject to the same cognitive demands of maintaining relationships that limit offline friendships. These constraints come in two principal forms: a cognitive constraint derivative of the SBH and a temporal constraint associated with the time that needs to be invested in a relationship to maintain it at a requisite level of emotional intensity [25]. We can only interact coherently with a very small number of other people (about three, in fact) at any one time [40,41]. It seems that even in an online environment, the focus of our attention is still limited in this way.

This conclusion is reinforced by analyses of the frequencies with which individuals communicate with members of their network in the inner network layers in online environments (Facebook and Twitter): these yield interaction rates that are virtually identical to those observed in the offline world [33]. Data from both face-to-face contacts [25,35] as well as mobile phone databases [34,77] suggest that there are natural limits to both the amount of time we can devote to social interactions with network members and how we distribute this time among them. Indeed, it seems that each of us distributes our social capital, whether this is indexed by frequency of calling or by self-rated emotional closeness, in a uniquely characteristic way rather like a social signature, and that this signature remains stable over time despite significant churn in network membership [77]. This appears to be immune to the opportunities for multiple interactions offered by the Internet.

Respondents were not asked to specify details about the individual alters in their networks (e.g. age, gender, emotional closeness or spatial proximity). Consequently, we cannot say whether participants’ decisions about whom to include as a Facebook friend reflect ease of access to individuals (e.g. how far away they lived, and so how easy it might be to meet up with them) or the distinction between family and friends (an important feature of face-to-face networks [22]). It may well be that the individuals in the sample with unusually small numbers of Facebook friends were using social media to maintain links only with distant family and friends. If so, this is only likely to affect the variance in the data by increasing the size of the left-hand tail (as figure 1 perhaps suggests). However, there is evidence to suggest that people do not use communication media only to contact geographically distant alters: to the contrary, mobile phone data show rather clearly that people in fact phone most frequently the people they live closest to [78].

The fact that people do not seem to use social media to increase the size of their social circles suggests that social media may function mainly to prevent friendships decaying over time in the absence of opportunities for face-to-face contact [76,79]. Given that people generally find interactions via digital media (including the phone as well as instant messaging and other text-based social media) less satisfying than face-to-face interactions [80], it may be that face-to-face meetings are required from time to time to prevent friendships, in particular, sliding down through the network layers and eventually slipping over the edge of the 150 layer into the category of acquaintances (the 500 layer) beyond. Friendships, in particular, have a natural decay rate in the absence of contact, and social media may well function to slow down the rate of decay. However, that alone may not be sufficient to prevent friendships eventually dying naturally if they are not occasionally reinforced by face-to-face interaction.

## Supplementary Material

Online Social Networks SI
