# Supplementary material for: Do online social media cut through the constraints that limit the size of offline social networks?
Source: R Soc Open Sci. 2016 Jan 20;3(1):150292. doi: 10.1098/rsos.150292 (PMC4736918; doi:10.1098/rsos.150292)
Supplement: Online Social Networks SI [file rsos150292supp1.docx]

**Do Online Social Media Cut Through the Constraints That Limit the Size of Offline Social Networks?**

**R.I.M. Dunbar**

**Supplementary Information**

The following tables summarise the data from the two samples (each of 2000 adults), from a structured sample of the UK proportionalised for regional distribution, age class and gender.

Supplementary Results are given below.

**Data**

**Survey 1**

Table S1. Respondents stated their total number of friends on Facebook on a categorical scale:

| # Facebook friends | **Female** | **Male** |
| --- | --- | --- |
| **0-25** | 14.40% | 21.04% |
|  | 158 | 190 |
| **26-50** | 12.12% | 15.17% |
|  | 133 | 137 |
| **51-75** | 12.49% | 13.73% |
|  | 137 | 124 |
| **76-100** | 12.40% | 9.30% |
|  | 136 | 84 |
| **101-200** | 21.79% | 17.61% |
|  | 239 | 159 |
| **201-300** | 11.76% | 9.97% |
|  | 129 | 90 |
| **301-400** | 5.38% | 5.87% |
|  | 59 | 53 |
| **401-500** | 4.28% | 2.21% |
|  | 47 | 20 |
| **501-600** | 2.01% | 1.66% |
|  | 22 | 15 |
| **601-700** | 0.73% | 1.11% |
|  | 8 | 10 |
| **701-800** | 0.55% | 0.44% |
|  | 6 | 4 |
| **801-900** | 0.55% | 0.44% |
|  | 6 | 4 |
| **901-1000** | 0.36% | 0.22% |
|  | 4 | 2 |
| **1001+** | 1.19% | 1.22% |
|  | 13 | 11 |
| **Average** | 165.45 | 144.99 |
|  |  |  |

Table 2. Respondents answered the question: “What percentage of your social media friends do you actually consider really genuine friends?”

Real friends Responses

(%) (%)

| 0-5 | 16.90% |
| --- | --- |
| 6-11 | 16.60% |
| 11-15 | 10.45% |
| 16-20 | 10.20% |
| 21-25 | 9.55% |
| 26-30 | 7.00% |
| 31-35 | 3.45% |
| 36-40 | 2.45% |
| 41-45 | 1.85% |
| 46-50 | 3.65% |
| 51-60 | 3.05% |
| 61-70 | 3.35% |
| 71-80 | 3.60% |
| 81-90 | 3.10% |
| 91-100 | 4.80% |

Table S3. Distribution of total network size by decadal age class:

Decadal age class:

| # Facebook friends: | **18-24** | **25-34** | **35-44** | **45-54** | **55+** |
| --- | --- | --- | --- | --- | --- |
| **0-25** | 8.45% | 6.82% | 15.49% | 25.16% | 37.40% |
|  | 18 | 45 | 70 | 77 | 138 |
| **26-50** | 8.92% | 8.94% | 11.50% | 18.63% | 22.49% |
|  | 19 | 59 | 52 | 57 | 83 |
| **51-75** | 9.86% | 10.30% | 14.16% | 18.30% | 14.09% |
|  | 21 | 68 | 64 | 56 | 52 |
| **76-100** | 8.45% | 13.18% | 13.50% | 8.17% | 7.86% |
|  | 18 | 87 | 61 | 25 | 29 |
| **101-200** | 13.15% | 27.27% | 23.01% | 15.69% | 10.30% |
|  | 28 | 180 | 104 | 48 | 38 |
| **201-300** | 14.55% | 16.21% | 10.18% | 6.54% | 4.07% |
|  | 31 | 107 | 46 | 20 | 15 |
| **301-400** | 10.33% | 8.18% | 5.31% | 1.96% | 1.63% |
|  | 22 | 54 | 24 | 6 | 6 |
| **401-500** | 9.39% | 3.94% | 2.21% | 1.96% | 1.36% |
|  | 20 | 26 | 10 | 6 | 5 |
| **501-600** | 3.76% | 2.73% | 1.99% | 0.33% | 0.27% |
|  | 8 | 18 | 9 | 1 | 1 |
| **601-700** | 4.23% | 0.61% | 0.88% | 0.33% | 0% |
|  | 9 | 4 | 4 | 1 | 0 |
| **701-800** | 1.88% | 0.30% | 0% | 1.31% | 0% |
|  | 4 | 2 | 0 | 4 | 0 |
| **801-900** | 1.88% | 0.76% | 0% | 0% | 0.27% |
|  | 4 | 5 | 0 | 0 | 1 |
| **901-1000** | 1.41% | 0% | 0.44% | 0.33% | 0% |
|  | 3 | 0 | 2 | 1 | 0 |
| **1001+** | 3.76% | 0.76% | 1.33% | 1.31% | 0.27% |
|  | 8 | 5 | 6 | 4 | 1 |
| **Average** | 282.05 | 185.68 | 150.01 | 114.56 | 73.01 |

Table S4. Respondents stated the total number of individuals in their support clique (those to whom they would go for support at times of great emotional or other distress) on a categorical scale:

| Support clique size | **Female** | **Male** |
| --- | --- | --- |
| **0** | 5.47% | 9.97% |
|  | 60 | 90 |
| **1 – 2** | 30.81% | 29.46% |
|  | 338 | 266 |
| **3 – 4** | 33.91% | 26.91% |
|  | 372 | 243 |
| **5 – 7** | 18.51% | 17.28% |
|  | 203 | 156 |
| **8 – 10** | 7.20% | 9.41% |
|  | 79 | 85 |
| **11 – 15** | 2.10% | 3.54% |
|  | 23 | 32 |
| **16+** | 2.01% | 3.43% |
|  | 22 | 31 |
| **Average** | 4 | 4.28 |

Table S5. Support clique size distribution by decadal age class:

Decadal age class:

| Support clique size: | **18-24** | **25-34** | **35-44** | **45-54** | **55+** |
| --- | --- | --- | --- | --- | --- |
| **0** | 7.51% | 6.52% | 6.64% | 6.54% | 11.11% |
|  | 16 | 43 | 30 | 20 | 41 |
| **1 - 2** | 31.46% | 24.39% | 30.75% | 34.31% | 35.77% |
|  | 67 | 161 | 139 | 105 | 132 |
| **3 - 4** | 29.58% | 31.97% | 29.42% | 32.68% | 29.27% |
|  | 63 | 211 | 133 | 100 | 108 |
| **5 - 7** | 16.43% | 23.03% | 18.58% | 14.38% | 11.92% |
|  | 35 | 152 | 84 | 44 | 44 |
| **8 - 10** | 8.45% | 9.24% | 8.41% | 7.52% | 6.50% |
|  | 18 | 61 | 38 | 23 | 24 |
| **11 - 15** | 3.29% | 2.73% | 3.54% | 2.29% | 1.90% |
|  | 7 | 18 | 16 | 7 | 7 |
| **16+** | 3.29% | 2.12% | 2.65% | 2.29% | 3.52% |
|  | 7 | 14 | 12 | 7 | 13 |
| **Average** | 4.21 | 4.39 | 4.25 | 3.86 | 3.67 |

Table S6. Respondents stated the number of close friends or acquaintances (sympathy group size) on a categorical scale:

| **# Close friends** | **%** |
| --- | --- |
| 0 | 2.5 |
| 1 - 2 | 8.05 |
| 3 - 4 | 14.95 |
| 5 - 10 | 32.5 |
| 11 - 15 | 13.6 |
| 16-20 | 13.55 |
| 21-30 | 6.1 |
| 31-40 | 3.05 |
| 41-50 | 2.35 |
| 51-75 | 1.35 |
| 76-100 | 0.6 |
| 101+ | 1.2 |
| Average | 13.61 |

Table S7. Sympathy group size distribution by decadal age class:

Decadal age class:

| Sympathy group size: | **18-24** | **25-34** | **35-44** | **45-54** | **55+** |
| --- | --- | --- | --- | --- | --- |
| **0** | 4.23% | 2.73% | 2.43% | 2.29% | 1.36% |
|  | 9 | 18 | 11 | 7 | 5 |
| **1 - 2** | 10.33% | 5.45% | 9.96% | 10.46% | 7.05% |
|  | 22 | 36 | 45 | 32 | 26 |
| **3 - 4** | 16.90% | 14.24% | 15.49% | 12.75% | 16.26% |
|  | 36 | 94 | 70 | 39 | 60 |
| **5 - 10** | 23.47% | 31.36% | 32.96% | 37.91% | 34.69% |
|  | 50 | 207 | 149 | 116 | 128 |
| **11 - 15** | 13.15% | 16.67% | 11.95% | 13.40% | 11.65% |
|  | 28 | 110 | 54 | 41 | 43 |
| **16-20** | 12.21% | 15.15% | 13.72% | 11.76% | 12.74% |
|  | 26 | 100 | 62 | 36 | 47 |
| **21-30** | 8.45% | 7.27% | 4.87% | 5.23% | 4.88% |
|  | 18 | 48 | 22 | 16 | 18 |
| **31-40** | 6.10% | 3.03% | 1.77% | 1.96% | 3.79% |
|  | 13 | 20 | 8 | 6 | 14 |
| **41-50** | 1.88% | 1.06% | 3.76% | 2.29% | 3.25% |
|  | 4 | 7 | 17 | 7 | 12 |
| **51-75** | 1.88% | 1.36% | 1.33% | 0.98% | 1.36% |
|  | 4 | 9 | 6 | 3 | 5 |
| **76-100** | 0.47% | 0.76% | 0.22% | 0% | 1.36% |
|  | 1 | 5 | 1 | 0 | 5 |
| **101+** | 0.94% | 0.91% | 1.55% | 0.98% | 1.63% |
|  | 2 | 6 | 7 | 3 | 6 |
| **Average** | 14.13 | 13.68 | 13.36 | 11.98 | 14.84 |

**Survey 2**

Table S8. Respondents were asked to state the total number of friends they had on Facebook to the nearest category:

| **# Facebook friends** | **Female** | **Male** |
| --- | --- | --- |
| **0** | **1.43%** | **3.02%** |
|  | **13** | **14** |
| **25** | **8%** | **18.57%** |
|  | **73** | **86** |
| **50** | **9.76%** | **13.39%** |
|  | **89** | **62** |
| **75** | **8.22%** | **10.37%** |
|  | **75** | **48** |
| **100** | **13.93%** | **11.02%** |
|  | **127** | **51** |
| **150** | **14.14%** | **12.10%** |
|  | **129** | **56** |
| **200** | **12.83%** | **9.50%** |
|  | **117** | **44** |
| **250** | **8.44%** | **5.18%** |
|  | **77** | **24** |
| **300** | **6.80%** | **4.97%** |
|  | **62** | **23** |
| **350** | **4.82%** | **3.24%** |
|  | **44** | **15** |
| **400** | **3.18%** | **1.30%** |
|  | **29** | **6** |
| **450** | **1.54%** | **1.30%** |
|  | **14** | **6** |
| **500** | **2.52%** | **1.73%** |
|  | **23** | **8** |
| **600** | **1.21%** | **1.51%** |
|  | **11** | **7** |
| **700** | **0.99%** | **1.08%** |
|  | **9** | **5** |
| **800+** | **2.19%** | **1.73%** |
|  | **20** | **8** |
| **Average** | **196.16** | **156.59** |
|  |  |  |

Table S9. Network size distribution by decadal age class:

Decadal age class:

| # Facebook friends: | **18-24** | **25-34** | **35-44** | **45-54** | **55+** |
| --- | --- | --- | --- | --- | --- |
| **0** | 0% | 0.75% | 2.38% | 5.05% | 2.99% |
|  | 0 | 4 | 9 | 10 | 4 |
| **25** | 1.52% | 3.38% | 11.11% | 20.71% | 41.79% |
|  | 2 | 18 | 42 | 41 | 56 |
| **50** | 4.55% | 5.63% | 12.96% | 19.70% | 20.15% |
|  | 6 | 30 | 49 | 39 | 27 |
| **75** | 1.52% | 8.07% | 10.58% | 12.12% | 10.45% |
|  | 2 | 43 | 40 | 24 | 14 |
| **100** | 9.09% | 15.01% | 12.96% | 14.65% | 5.97% |
|  | 12 | 80 | 49 | 29 | 8 |
| **150** | 9.85% | 15.38% | 16.40% | 10.61% | 5.22% |
|  | 13 | 82 | 62 | 21 | 7 |
| **200** | 16.67% | 16.51% | 10.58% | 3.54% | 2.99% |
|  | 22 | 88 | 40 | 7 | 4 |
| **250** | 6.06% | 9.38% | 7.94% | 3.54% | 4.48% |
|  | 8 | 50 | 30 | 7 | 6 |
| **300** | 8.33% | 7.32% | 5.29% | 6.06% | 2.24% |
|  | 11 | 39 | 20 | 12 | 3 |
| **350** | 11.36% | 5.07% | 3.17% | 1.52% | 1.49% |
|  | 15 | 27 | 12 | 3 | 2 |
| **400** | 7.58% | 3% | 2.12% | 0.51% | 0% |
|  | 10 | 16 | 8 | 1 | 0 |
| **450** | 5.30% | 2.06% | 0.26% | 0.51% | 0% |
|  | 7 | 11 | 1 | 1 | 0 |
| **500** | 3.79% | 3.38% | 1.59% | 0.51% | 0.75% |
|  | 5 | 18 | 6 | 1 | 1 |
| **600** | 3.03% | 1.69% | 1.06% | 0% | 0.75% |
|  | 4 | 9 | 4 | 0 | 1 |
| **700** | 4.55% | 0.94% | 0.79% | 0% | 0% |
|  | 6 | 5 | 3 | 0 | 0 |
| **800+** | 6.82% | 2.44% | 0.79% | 1.01% | 0.75% |
|  | 9 | 13 | 3 | 2 | 1 |
| **Average** | 318.56 | 218.34 | 158.6 | 108.96 | 85.45 |
|  |  |  |  |  |  |

**Supplementary Results**


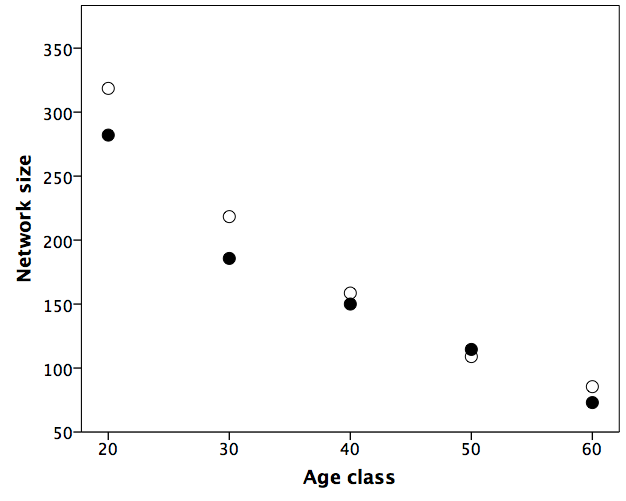


Fig. S1.

Mean total network size for each decadal age class in the two samples.

Filled circles: Sample 1; open circles: Sample 2.


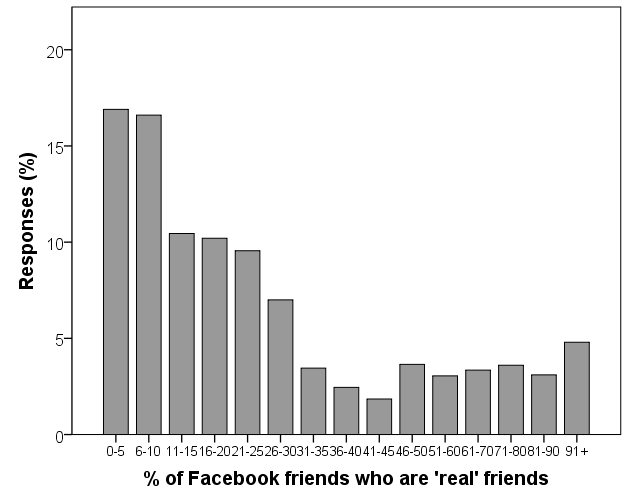


Fig. S2.

Percent of respondents in Sample 1 who considered different proportion of their Facebook friends to be ‘genuine’ friends.
